# Modification of 3D printing resin with surface treated mesoporous silica nanoparticles: structural and mechanical characterization

**DOI:** 10.1186/s12903-026-07993-5

**Published:** 2026-03-24

**Authors:** Mohamed S. Morad, Manal Ahmed El-Ebiary, El-Refaie Kenawy, Enas A. Elshenawy

**Affiliations:** 1 Dental Biomaterials Department, Faculty of dentistry, Tanta University, Tanta, 31773 Egypt; 2 Chemistry Department, Faculty of Science, Tanta University, Tanta, 31111 Egypt

**Keywords:** 3D printed resins, Mesoporous silica, Nanoparticles, Grafting Reaction, Surface modification, Provisional restorations

## Abstract

**Background:**

This study aims to augment the advantages of three-dimensional (3D) printed provisional restoration with a higher weight% (wt%) of nanofillers through evaluating the effect of simultaneous silanization and grafting reaction on the surface of mesoporous silica nanoparticles (MSN) and testing the effect of modification of 3D-printable provisional resin (3D-PPR) with surface treated MSN on its physicomechanical properties.

**Methods:**

The surface of MSN was first silanized with (3-Aminopropyl) triethoxysilane (APTES) and then silanized MSN was grafted with methyl methacrylate (MMA). The surface modifications of MSN were characterized by transmission electron microscopy (TEM), Brunauer–Emmett–Teller (BET) surface area, Barrett–Joyner–Halenda (BJH) pore size analyses, fourier transformed infrared spectroscopy (FTIR) and thermogravimetric analysis (TGA). Modified 3D-PPR specimens with 0.25, 1 and 3 wt% of silanized MSN (S-MSN) and grafted silanized MSN (GS-MSN) were printed and evaluated by degree of conversion, surface morphology, surface roughness, surface hardness, flexural strength, flexural modulus and fracture resistance.

**Results:**

The simultaneous surface treatment of MSN decreased their agglomeration and enhanced their distribution within 3D-PPR. The modified 3D-PPR with GS-MSN showed a gradual enhancement in physicomechanical properties by increasing filler wt%, in contrast to modification with S-MSN only which showed a gradual decrease in physicomechanical properties by increasing filler wt%.

**Conclusion:**

This simultaneous surface treatment of MSN opens the door for increasing MSN wt% into 3D-PPR to have the superior potential and efficacy for long-term provisional dental restorations.

**Graphical Abstract:**

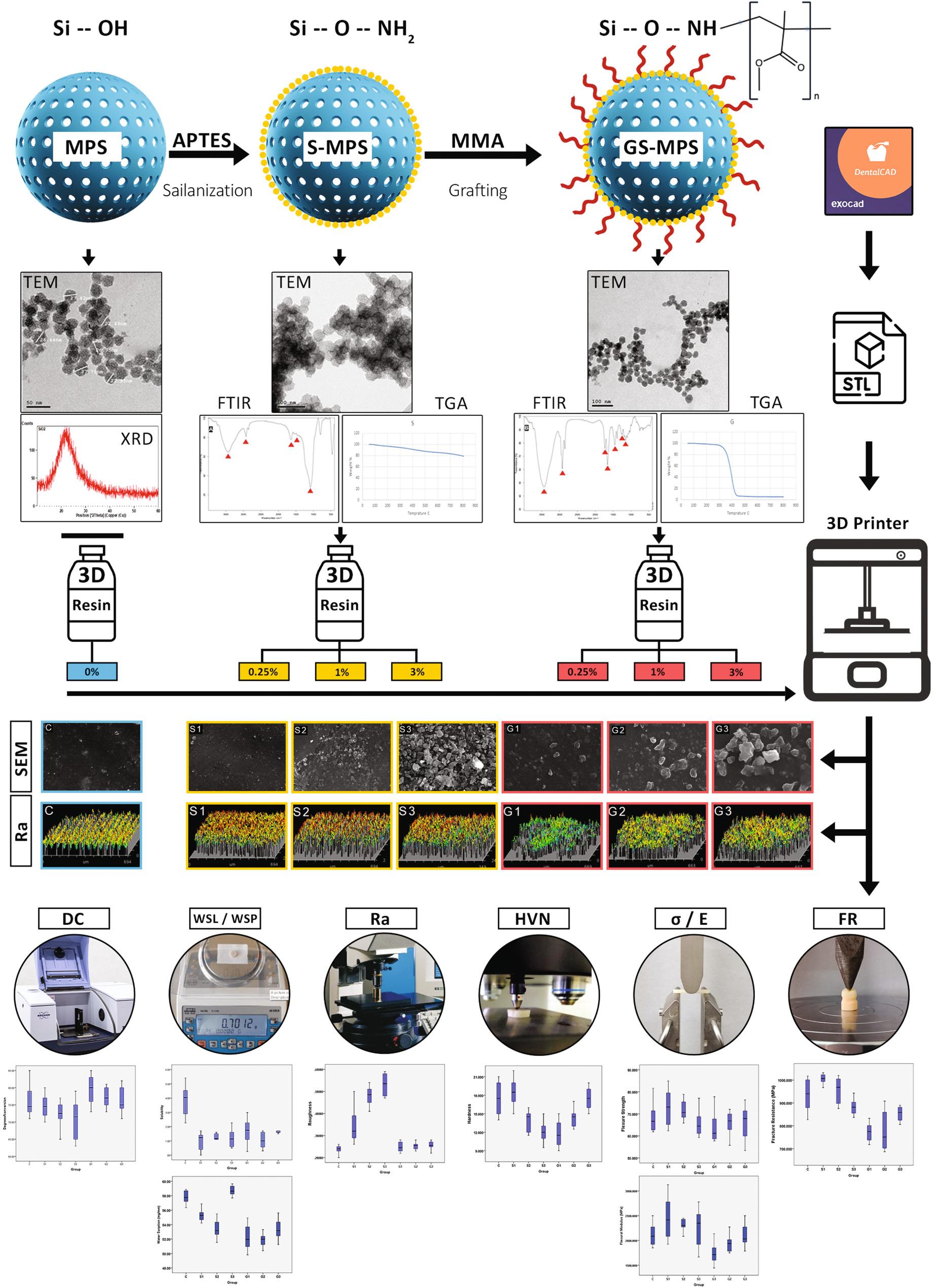

## Background

In fixed prosthodontics, the performance of provisional restorations is essential to the success of the final prostheses [1]. Provisional restorations are much more than just temporary restorations as one can argue that they contribute significantly to the patient’s health and satisfaction. They ensure appropriate occlusion, maintain vertical distance, improve aesthetics, and restore function [2]. Furthermore, provisional prostheses ought to protect the prepared teeth from mechanical and thermal stresses and promote gingival healing specially with in implant-supported prosthesis [3]. Therefore, when selecting provisional materials, it is essential to consider all the other important criteria such as high physicomechanical properties, adequate biocompatibility, convenience in handling, and cost efficiency of the material [4].

Additive manufacturing, also known as three-dimensional (3D) printing or rapid prototyping, has revolutionized the field of modern dentistry by producing highly accurate 3D printed restorations and prostheses with relatively economic advantages. Photo-curable 3D printable resin has been used in producing surgical guides, diagnostic models, custom tray, complete dentures, splints, and provisional restorations [5, 6]. Provisional restorations are commonly manufactured using resin-based materials, which are homogenized combination of filler particles and resin matrix mostly polymethyl methacrylate (PMMA) [7]. PMMA resins are an economic choice, have good color stability, good marginal accuracy, and polishability. However, the main drawbacks of this type of resin under function are water sorption, low fracture strength and low wear resistance, which may cause clinical failure [8, 9].

Consequently, efforts have been made to enhance performance and longevity of provisional modified resins. Most of these recent efforts have been directed towards modifying the resin matrix by incorporating different organic and inorganic fillers that create modified resins with enhanced properties [10, 11]. Previous studies have indicated that modifying dental resins with different micro and nanoscale fillers significantly enhanced their physicomechanical properties [12, 13]. Conversely, some of these modifications have resulted in adverse effects that compromise material performance, such as creation of voids, limiting depth of cure and degree of polymerization [14–17].

Silica nanoparticles (SN) in particular, mesoporous silica nanoparticles (MSN) are widely used in acrylic-based dental resin to achieve the desired mechanical properties with adequate biocompatibility, durability, and high structural stability [18–22]. Nonetheless, incorporating more than 1% by weight resulted in negative effects on physicomechanical properties. This was attributed to the formation of agglomerates of nanoparticles at elevated weight% (wt%), leading to the creation of loosely bonded clusters. These clusters serve as focal points for stress, causing cracks to begin and spread in these regions [1, 23–27].

Having a porous structure, MSN functions efficiently as a drug carrier to store, deliver, and release various antimicrobial agents. However, it would be beneficial if added with higher concentration without agglomeration. Specially for implant-supported prosthesis, owing to complex osseointegration mechanisms and gingival healing thanks to its porous structure [3, 18, 22, 28]. Liao et al. [29] concluded that chemical surface modifications of silica nanoparticles prevent their agglomeration within the organic matrix. Silane coupling agent treatment and surface grafting modification were the two most applied strategies to overcome those problems. To our knowledge, there is still a scarcity of research focused on the simultaneous surface silanization and grafting reaction on the surface of SN or MSN and its applications in the dental field.

The objectives of this study were: (i) To augment the advantages of 3D printing technology with adding a higher percentage of MSN to a 3D-printable provisional resin (3D-PPR). (ii) To evaluate the effect of simultaneous silanization and grafting reaction on the surface of MSN on their agglomeration. (iii) To test the effect of modification of 3D-PPR material with surface treated MSN, at different wt%, on its physicomechanical properties.

The null hypotheses tested were: (i) Simultaneous silanization and grafting reaction on the surface of MSN has no effect on their agglomeration. (ii) Modifying 3D-PPR with surface treated MSN, at different percentage, has no effect on its physicomechanical properties.

## Materials and methods

The materials that were used in this study are presented in Table 1.

**Table 1:** Materials used in the study

Category	Composition	Manufacturer/ Supplier
Nanoparticles MSN	- Mesoporous silica nanoparticles < 50 nm	Nano Gate, Mokattam, Cairo, Egypt
Silane coupling agent	− (3-Aminopropyl) triethoxysilane (APTES)	Sigma Aldrich, St. Louis, MO, USA
Reagents for silanization	- Anhydrous ethanol	Sigma Aldrich, St. Louis, MO, USA
Grafting Monomer	- Methyl methacrylate (MMA)	Sigma Aldrich, St. Louis, MO, USA
Reagents for grafting	- Xylene - Benzoyl peroxide (BPO) - Acetone	Sigma Aldrich, St. Louis, MO, USA
3D-printable resin for temporary crown and bridge	− 405 nm UV curable resin, mainly methyl methacrylate (MMA)	(Proshape temp resin, Proshape Dental Solutions, Turkey)

Sample size was calculated using a computer program G power version 3 based on a previous study with 95% power and effect size of 0.35, resulting in a minimum sample size 10/test/group [30] which was sufficient to detect statistically significant differences among the tested groups. The significance level was 0.05. A total number of three hundred fifty (350) specimens was classified and tested according to the grouping presented in Table 2.

**Table 2:** Experimental study design illustrating classification of all groups

Modifications	Groups	Characterization/Tests
Control without mesoporous silica	Group C, *n* = 50 0 wt% fillers	A. Characterization 1. Scanning electron microscopy (SEM). 2. Transmission Electron microscopy (TEM) 3. Fourier transformed infrared spectroscopy (FTIR) 4. X-Ray diffraction analysis (XRD) 5. Thermogravimetric analysis (TGA) 6. Brunauer–Emmett–Teller theory (BET) 7. Barrett–Joyner–Halenda (BJH) B. Physicomechanical assessment 1. Degree of conversion (DC) 2. Solubility and water sorption (WSL & WSP) 3. Surface roughness (Ra) 4. Surface microhardness (HVN) 5. Flexure strength (σ) 6. Flexure modulus (E) 7. Fracture resistance (FR)
Experimental with Silanized mesoporous silica	Group S1, *n* = 50 0.25 wt% fillers	
	Group S2, *n* = 50 1 wt% fillers	
	Group S3, *n* = 50 3 wt% fillers	
Experimental with Grafted Silanized mesoporous silica	Group G1, *n* = 50 0.25 wt% fillers	
	Group G2, *n* = 50 1 wt% fillers	
	Group G3 *n* = 50 3 wt% fillers	

### Specimens preparation

MSN were functionalized with amino groups (NH_2_) by treating with (3-Aminopropyl) triethoxysilane (APTES). MSN (1 g) was added into 200 ml anhydrous ethanol and the solution was stirred for 5 min. Then 0.5 ml APTES was added, and the solution was stirred for 8 h at 80 °C. The surface silanized mesoporous silica nanoparticles (S-MSN) were collected by centrifugation, washed with double-distilled water several times [31].

S-MSN were grafted with methyl methacrylate (MMA) using free radical polymerization. S-MSN (1 g) was dispersed in 50 g xylene in a 250mL four-neck flask with a machine stirring and ultrasonically dispersed for 0.5 h. Afterward, 0.1 g benzoyl peroxide (BPO) initiator and 10 g MMA were added to the system under nitrogen atmosphere. The mixed suspension was kept stirring and heated to 80 °C in the water bath. After 8 h polymerization, the grafted silanized mesoporous silica nanoparticles (GS-MSN) were obtained through centrifugal separation and extraction with acetone to remove any free MMA and separate any free PMMA, then dried in the vacuum oven at 80 °C for 12 h [19, 29, 32].

A series of modified 3D-PPR was prepared with different percentages of S-MSN and GS-MSN as listed in Table 2. The S-MSN and GS-MSN were added to the 3D-PPR (Proshape temp resin, Proshape Dental Solutions, Turkey) under continuous magnetic stirring (MS300HS, Miseong Science Equipment, Korea) for 1 h, followed by sonication (Power Sonic 405, Whashin Co., Korea) for 10 min operated with 250 W to obtained well-dispersed modified 3D-PPR ready for the 3D printing [18, 29].

For 3D printing, a Digital Light Processing (DLP) 3D printer (RASDENT 3D printer, RASPART, Egypt) was used. Different sizes and geometries of specimens were designed by Exocad software (Exocad Dental IDB 2.4 plovdiv7290, version 2.4 Engine build 7290, Exocad GmbH, Darmstadt, Germany) according to the specifications of each test. The generated stereolithography (STL) files were sent to the 3D printer. The printing parameters, printing orientation (0° horizontally), job resolution for each layer (50 μm thickness), exposure time per layer (5 s.), UV light wavelength (405 nm) and Pulse Width Modulation (255), were maintained constant for all specimens in accordance with the manufacturer’s predefined settings to eliminate confounding variables and ensure standardization. The printed specimens were scrapped off from the printing stage, cleaned, and sonicated twice with isopropanol for 5 min to remove any excess uncured resin, then rinsed twice with distilled water. To reach maximum strength, they were exposed to a post-curing step through 5 min curing in a curing unit at 405 nm wavelength (Light Zone II DS-310, Denstar, South Korea). The effectiveness of monomer removal was confirmed based on the manufacturer’s recommended protocol and supported by the FTIR analysis before and after curing. Finally, the specimens were cleaned, finished and polished according to the manufacturer’s recommendations. All specimens were stored in distilled water in an incubator (BTC, BT1120, Biotech, Cairo, Egypt) at 37 °C for 24 h before testing [33].

### Characterization

#### X-ray diffraction (XRD) for MSN

The crystalline nature was analyzed using XRD (XRD, Diffractometer, Model: GNR APD-2000 PRO, Toryno, Italy). An XRD pattern has been performed using XPERT-PRO Powder Diffractometer system. The data were captured from 10° to 60° using a 0.02 θ step size at wavelength (Kα) = 1.54614ᴼ and a 0.3°/min scanning speed. It used a spinning x-ray generator with 40 kW and 40 mA [21, 34].

#### Transmission electron microscopy (TEM) for MSN, S-MSN and GS-MSN

Morphology and microstructure were characterized by TEM (TEM, JEM-2100 F, JEOL, Japan) at an accelerating voltage of 200 kV. Each sample powder for TEM was prepared by placing a droplet of colloid suspension in respective solvent on a Formvar carbon-coated, 300-mesh copper grid (Ted Pella) and allowing them to evaporate in air at ambient conditions.

#### Nitrogen adsorption–desorption measurements for S-MSN and GS-MSN

The surface area, pore diameter and pore volume were estimated using Brunauer-Emmett-Teller (BET) and Barrett–Joyner–Halenda (BJH), using nitrogen adsorption–desorption analysis measured on a Microtrac Belsorp miniX apparatus (BELSORP-miniX, Microtrac BEL Corp., Japan) S/N 10039, Software version 1.1.3.1.

#### Fourier transform infrared spectroscopy (FTIR) for S-MSN and GS-MSN

Chemical composition was characterized by FTIR Tensor 27 Bruker spectrometer (Bruker Optik GmbH, Germany). Each sample powder was pressed into a pellet with potassium bromide. FTIR spectra were recorded in scanning range (4000 to 400 cm-1), spectral resolution (4 cm-1) and number of scans (16–64 scan/sec).

#### Thermogravimetric analysis (TGA) for S-MSN and GS-MSN

Thermal stability of S-MSN and GS-MSN was determined using a thermogravimetric analyzer (PerkinElmer, TGA4000, USA) balance type top-loading balance with temperature range Ambient to 1000˚C. Scanning rates 0.1 to 200˚C/min. The samples (3–5 mg) were taken in standard alumina cups and an empty cup was used as a reference. The TGA thermogram was obtained by heating the samples from 50 to 800˚C with a heating rate of 10˚C/min under a nitrogen flow of 20 ml/min. Grafting amount and percentage of MMA onto S-MSN was investigated by the following equation [19].

$$Grafted\;amount\;(mequiv/g) = [1000\;x\;W]\;/\;[(100-W)\;x\;M]$$

In this equation, W is the difference between the weight losses of S-MSN before and after grafting in the range of 200–800̊C and M is the molecular weight of the MMA.

#### Scanning electron microscopy (SEM) for modified 3D-PPR

Dispersion of nanoparticles and surface morphology were characterized by SEM (JEOL-JSM-6510LV, Tokyo, Japan). Gold was sputter deposited onto the samples. Then, magnifications ranging from 1000 to 5000 times were used to inspect the samples.

### Testing methods

#### Degree of conversion (DC)

FTIR was used to determine the effect of adding S-MSN and GS-MSN on the degree of polymerization. In the FTIR spectra, two characteristic bands at 1637 cm− 1 (stretching of carbon double bond C = C) and 1720 cm− 1 (stretching of carbonyl group C = O) before and after polymerization for all groups were used to calculate the degree of conversion using the following equation [18].

$$DC\;(\%)\;=\;(1- [A(C\;=\;C)/A(C\;=\;O) polymer / A(C\;=\;C)/A(C\;=\;O)] monomer) x100.$$

where A(C = C) and A(C = O) are the band intensities.

#### Solubility and water sorption (WSL / WSP)

According to the International Standards Organization ISO 10477:2020, [35] 10 disc shaped specimens with a diameter of 15 mm and thickness of 1 mm were printed for each group. Specimen volume (V) was measured using a digital caliper (RS Pro, Corby, UK), then specimens were kept under dry conditions in a desiccator containing silica granules in an incubator (BTC, BT1120, Biotech, Cairo, Egypt) at 37˚C for seven days. Then specimens were taken out and weighed using an analytical balance with an accuracy of 0.001 g (RADWAG Wagi Elektroniczne, Poland) for (m1). The dried specimens were immersed in distilled water in an incubator at 37˚C for seven days. After that, specimens were removed, wiped with absorbing paper, and weighed again (m2). The immersed specimens were dried again for seven days as mentioned before. Then specimens were taken out and weighed again for (m3). The following equations were used to calculate water sorption (WSP) and solubility (WSL) [36].

$$\mathrm{WSP} = (\mathrm{m}2 - \mathrm{m}3)/\mathrm{V} (\mathrm{ug/mm}^{3})$$

$$\mathrm{WSL} = (\mathrm{m}1 - \mathrm{m}3)/\mathrm{V} (\mathrm{ug/mm}^{3})$$

Where: m1 = sample weight in mg before immersion, m2 = sample weight in mg after immersion, m3 = sample weight in mg after immersion and desiccation, V = specimen volume in mm^3^.

#### Surface roughness (Ra, µm)

The evaluation of the surface roughness was carried out using a non-contact optical profilometer (ZYGO MaximGP200, USA), which is a general-purpose surface optical profiler that measures the microstructure and topography of surfaces in three dimensions. The scanning white light interferometry provides advanced surface texture software which analyzes areas as well as profiles and step height. A 20X Mirau objective has been used in the measurements. The average roughness (Ra) value for each specimen was calculated by averaging three readings taken from various locations on the surface.

#### Surface microhardness (VHN)

The surface microhardness was measured and reported as Vickers hardness number (VHN) using microhardness tester (Zwick/Roell, INDENTEC, ZHVµ-S, West Midlands, England). Each specimen was subjected to a diamond indenter with a load force of 50 g and dwell time of 15s. Three indentations were made on the surface of each specimen. The diagonals length of the indentations was viewed by 10X objective lens and measured with a built-in scaled microscope. The hardness number was calculated with the machine’s software.

#### Flexural strength and flexural modulus (σ / E)

According to ISO 10477:2020, [35] flexural strength specimens (25 mm × 2 mm × 2 mm) were printed for each group. Each specimen was subjected to three point bending test using a Universal Testing Machine (Instron Model 3365; Instron Corp., Canton, MA, USA) at a crosshead speed of 1.0 mm/min until the specimen fractured. The flexural strength (σ) and flexural modulus (E) were calculated in MPa according to the following formula [37].

$$\upsigma = 3\mathrm{FL}\, /\, 2\mathrm{bh}^{2}$$

$$\mathrm{E} = \mathrm{F}_{1}\mathrm{L}^{3}\, /\, 4\mathrm{bh}^{3}\mathrm{d}$$

Where F is the maximum applied load (N); L is the distance (mm) between the supports; b is the width of the test specimen (mm); h is the height of the specimen (mm); F_1_ is the load (N) at a point in the straight-line portion of the load/deflection curve; and d is the deflection (mm) at load F_1_.

#### Fracture resistance (FR)

A die and a full contour lower molar crown were designed according to ISO 4049:2019, [33] then printed out for each group. Resin luting cement (G-Cem, GC Corporation, Tokyo, Japan) was used for luting the crowns. After removal of excess cement all specimens were light cured for 20s and stored in distilled water at 37 °C for 24 h. Then each specimen was loaded by a metal stylus with a 5 mm-diameter spherical tip applied directly to the center of each crown and perpendicular to the occlusal surface until fracture using a universal testing machine (Instron Model 3365; Instron Corp., Canton, MA, USA) at a crosshead speed of 1.0 mm/min. Maximum applied load (N) was recorded for each specimen [38].

### Statistical analysis

Data was collected, and statistical analysis was performed using IBM SPSS software for windows. Shapiro-wilk test was performed to test normality of data. Being normal, analysis of variance (ANOVA) was used to determine the variance between the different groups. Tukey’s test was used for pair-wise comparisons if needed. For DC, the Kruskal-Wallis test followed by Mann-Whitney U test was performed. The significance level will be set at *P* ≤ 0.05. Spearman’s correlation test was performed to detect if there is a significant relation between both filler fractions and tested properties.

## Results

### Morphological and chemical characterization

**Fig. 1:**
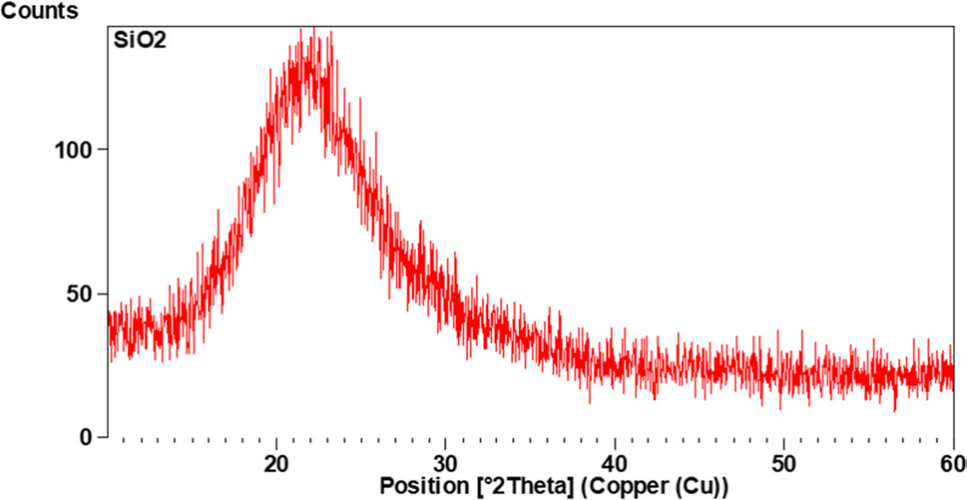
XRD patterns of MSN showing the characteristic peak at 2θ = 22–24 indicating amorphous structure

XRD diagram Fig. 1 showed the broad peak at around 2θ = 22–24° was attributed to the amorphous silica phase. This amorphous-like pattern corresponds to the formation of porous structure in the material.

**Fig. 2:**
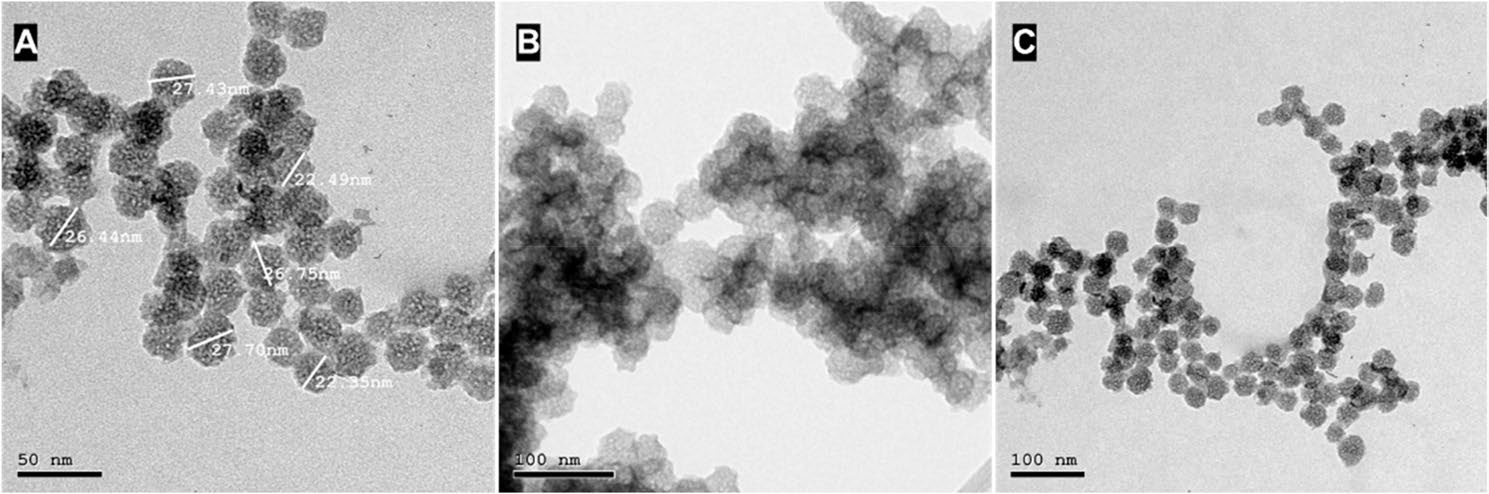
TEM images showing texture and particle size of (**A**) MSN and Agglomeration of (**B**) S-MSN (**C**) GS-MSN

Acquired images from TEM showed that MSN have a uniform spheroidal shape with particle size less than 50 nm and contained nanopores as shown in Fig. 2A. TEM images of S-MSN and GS-MSN showed that silanization and grafting did not affect the basic structure of nanoparticles, other than forming a thin coating around them as shown in Fig. 2B, C. But there was less agglomeration of the GS-MSN (Fig. 2C) as compared with S-MSN (Fig. 2B).

Nitrogen adsorption–desorption analysis (BET/BJH) as shown in Table 3 demonstrated that S-MSN exhibited BET surface area of 158 m²/g and a total pore volume of 0.506 cm³/g with BJH average pore diameter of 12.81 nm. MMA grafting (GS-MSN) resulted in a pronounced decrease in surface area (2.75 m²/g) and pore volume (0.033 cm³/g) with apparent increase in average pore diameter to 47.71 nm.

**Table 3:** Nitrogen adsorption–desorption analysis of S-MSN and GS-MSN

Particles	BET surface areas (m^2^/g)	BET pore volume (cm^3^/g)	BJH average pore diameter nm
S-MSN	158	0.506	12.81
GS-MSN	2.75	0.033	47.71

**Fig. 3:**
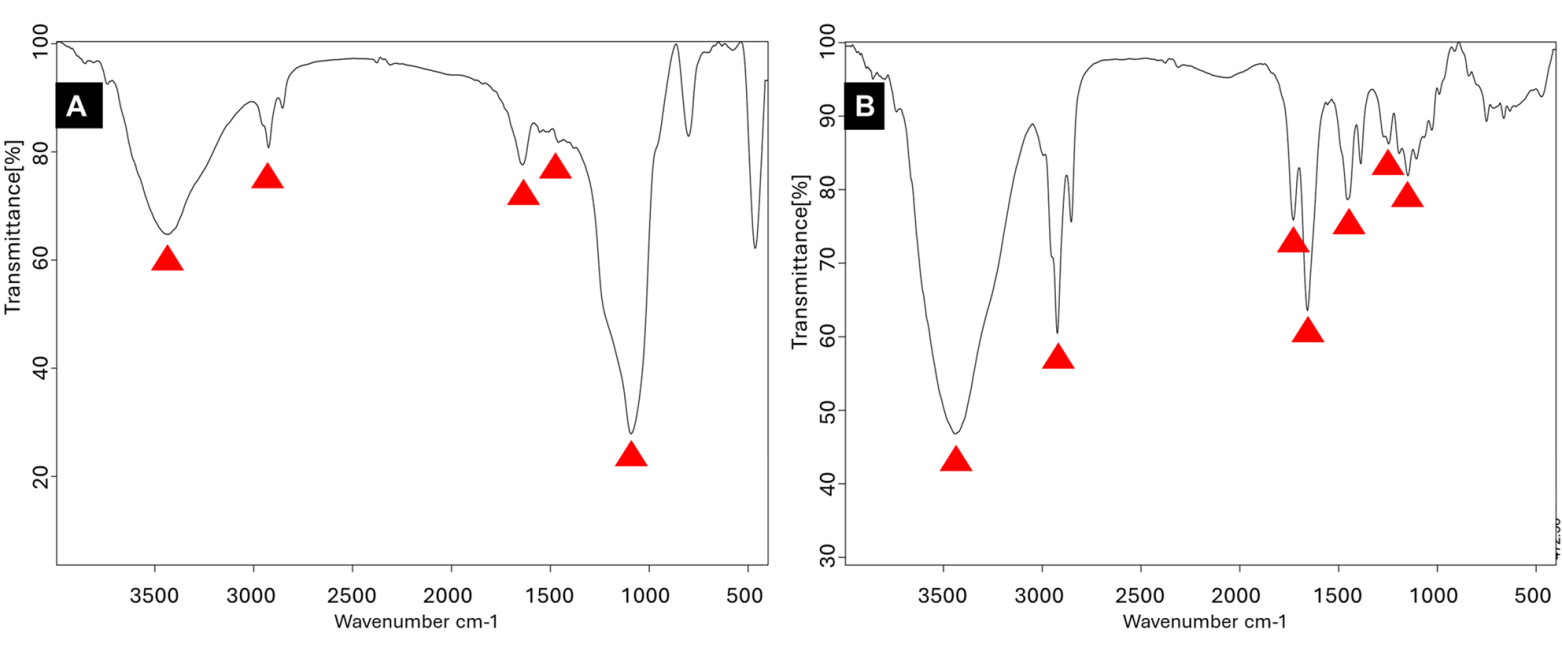
FTIR spectroscopy reflects characteristic peaks of (**A**) S-MSN and (**B**) GS-MSN

From the FTIR spectra of S-MSN as shown in Fig. 3A, a wide absorption peak can be observed at 1082 cm^− 1^. The characteristic peaks around 1638 cm^− 1^ and 1679 cm^− 1^ were observed. Several minor bands at around 2800 to 3000 cm^− 1^ were also detected. From the FTIR spectra of GS-MSN as shown in Fig. 3B, absorption bands can be observed at 3439 cm^–1^, 2929 cm^–1^, 1720 cm^–1^, 1390 cm^–1^, 1246 cm^–1^ and 1150 cm^–1^. The absorption bands around 1717 cm^–1^ are collapsed bands.

**Fig. 4:**
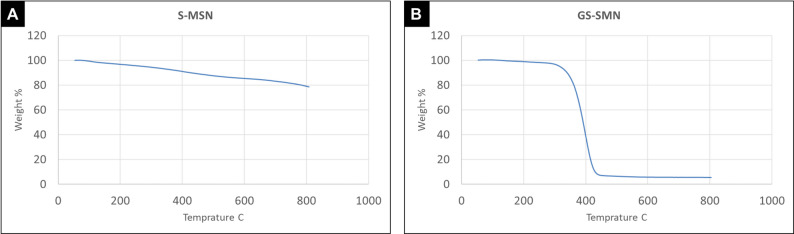
TGA thermograms are showing the percentage weight loss of (**A**) S-MSN and (**B**) GS-MSN with the increase in temperature

In the TGA curve of S-MSN as shown in Fig. 4A, there was a little weight loss below 200 °C then there was a second stage of a little more weight loss was observed in the range of 200–800 °C. In the TGA thermogram of GS-MSN as shown in Fig. 4B, a small weight loss occurred before 200 °C. In the next step, a large weight loss was observed in the range of 280–400 °C. According to the difference between the weight losses of S-MSN and GS-MSN at the temperature range of 200–800 °C, the amount and percentage of the bonded MMA on the surface of S-MSN was obtained from the previously mentioned equation as 31wt%. Consequently, the inorganic filler fraction inside each filler wt% was calculated as shown in Table 4.

**Table 4:** The inorganic and organic filler fraction inside each filler wt% for each group

Group Filler wt%	Inorganic filler Fraction wt%	Organic filler Fraction wt%
C (0 wt%)	0	0
S1 (0.25 wt%)	0.25	0
S2 (1 wt%)	1	0
S3 (3 wt%)	3	0
G1 (0.25 wt%)	0.1725	0.0775
G2 (1 wt%)	0.69	0.31
G3 (3 wt%)	2.07	0.93

**Fig. 5:**
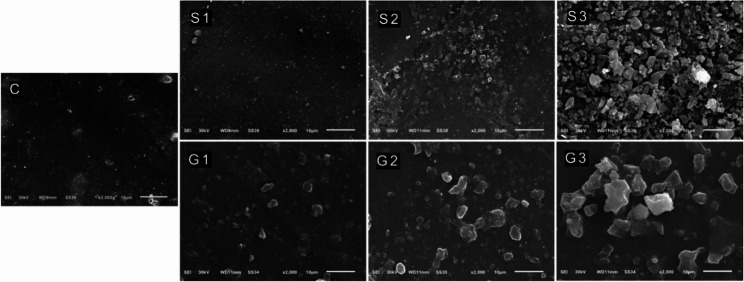
SEM micrographs showing the differences in fillers distribution and agglomeration on the surfaces of all groups

From the SEM images as shown in Fig. 5, the experimental groups with S-MSN, showed a clear increase in nanoparticles agglomeration and decrease in their distribution by increasing the filler wt%. The same was noticed for experimental groups with GS-MSN but to a lesser degree specially at G3 as compared with S3 and both with the highest filler wt%.

### Testing results

**Table 5:** Demonstrate mean, standard deviation, F and P-Value of physicomechanical properties among all groups

	C	S1	S2	S3	G1	G2	G3	F	*P*-Value
WSL(ug/mm3)	3.8 ± 1.06	1.03 ± 0.58	1.13 ± 0.48	1.14 ± 0.6	1.66 ± 0.88	1.04 ± 0.54	1.62 ± 0.06	22.181	0.000*
WSP (ug/mm3)	57.8 ± 0.87	55.3 ± 0.82	53.4 ± 1.23	58.7 ± 0.71	52.1 ± 1.64	51.8 ± 0.94	53.4 ± 1.31	59.010	0.000*
Ra (um)	0.24 ± 0.02	0.34 ± 0.07	0.48 ± 0.04	0.52 ± 0.06	0.25 ± 0.02	0.26 ± 0.02	0.26 ± 0.02	92.362	0.000*
HVN	17.5 ± 2.5	18.6 ± 2.1	14 ± 1.7	12.1 ± 1.7	11.8 ± 2	14.7 ± 1.9	17.5 ± 1.6	19.718	0.000*
σ (MPa)	68.4 ± 6.9	73 ± 8.2	71.3 ± 4.2	65.6 ± 5.4	63.8 ± 6.5	65.5 ± 5.5	66.1 ± 7.7	2.745	0.020*
E (MPa)	2134 ± 234	2427 ± 389	2336 ± 199	2254 ± 372	1740 ± 207	1945 ± 160	2080 ± 214	7.850	0.000*
FR (N)	931.2 ± 71	988.1 ± 54	953.9 ± 62	882.2 ± 32	774.6 ± 38	784.2 ± 89	852.6 ± 31	26.668	0.000*

An asterisk (*) indicates a statistically significant difference (*p* < 0.05)

#### Degree of conversion (DC)

**Fig. 6:**
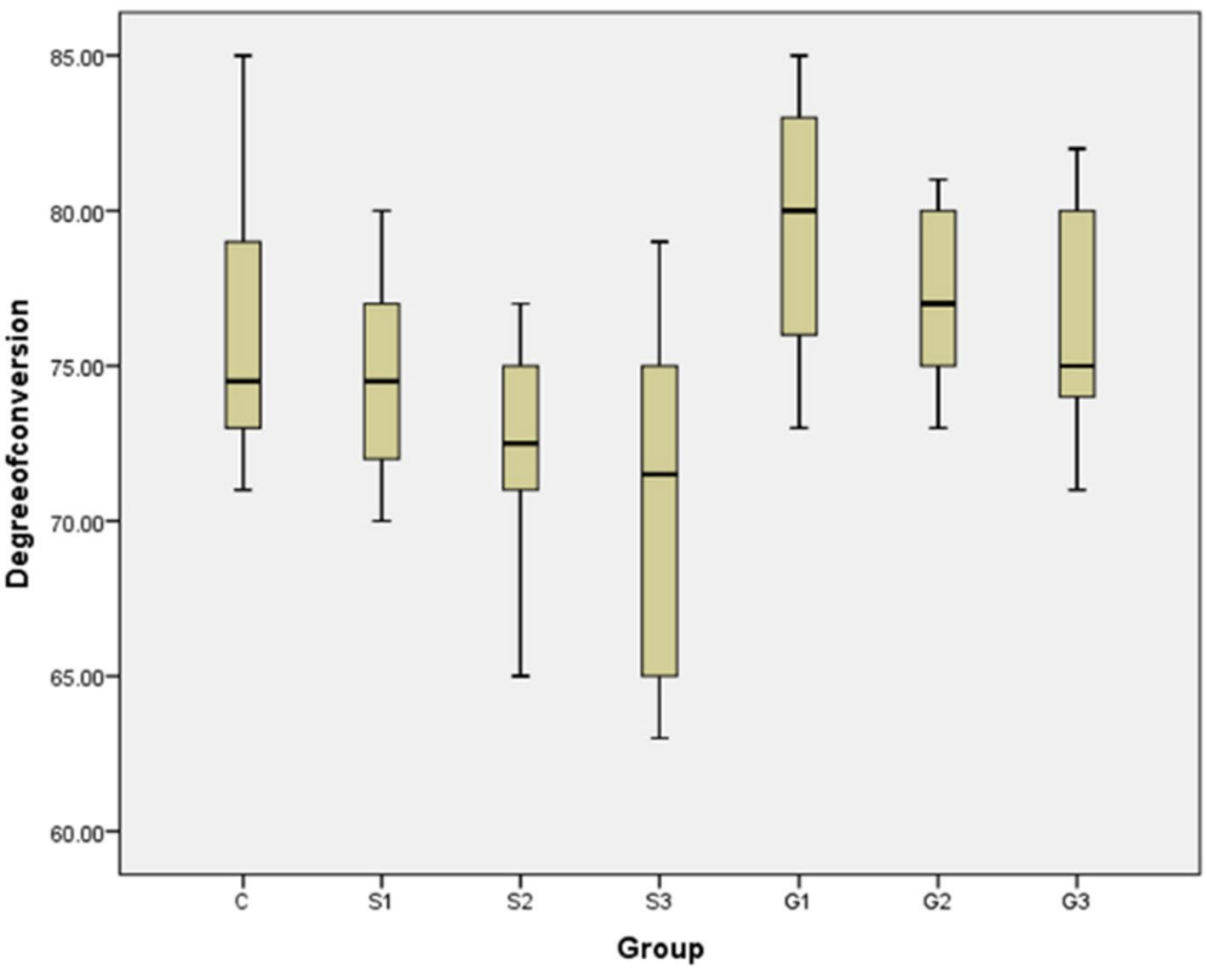
Graph demonstrating DC of unmodified printed resin and modified printed resin with S-MSN and GS-MSN 0.25,1,3%

Degree of Conversion (DC) of all tested groups means ± standard deviations values were presented in Fig. 6. The highest mean value was recorded for G1 (79.7 ± 4.2). The lowest value was recorded for S3 (70.8 ± 5.8). The Kruskal-Wallis test showed the statistically significant differences between the groups (*p* = 0.003*). Mann-Whitney U test showed that there were no significant differences between the control group (75.9 ± 4.6) and all experimental groups. But there was a slight non-significant decrease (*p* > 0.05) by increasing filler wt% in S groups (S1: 74.7 ± 3.2; S2: 72.4 ± 3.4; S3: 70.8 ± 5.8) and G groups (G1: 79.7 ± 4.2; G2: 77.1 ± 2.9; G3: 76.1 ± 3.7). On the other hand, there was a significant increase (*p* < 0.05) in each G group when compared with the corresponding S group.

#### Solubility (WSL)

Solubility values (µg/mm^3^) of all tested groups means ± standard deviations values were presented in Table 5. The highest WSL mean value was recorded for C (3.8 ± 1.06). The lowest value was recorded for S1 (1.03 ± 0.58). One-way ANOVA test showed the statistically significant differences between the groups (*p* = 0.000*). Tukey post-hoc test showed that there was a significant decrease in all S and G groups as compared with C group and there were no significant differences between all experimental groups.

#### Water sorption (WSP)

Water sorption values (µg/mm^3^) of all tested groups means ± standard deviations values were presented in Table 5. The highest WSP mean value was recorded for S3 (58.7 ± 0.71). The lowest value was recorded for G2 (51.84 ± 0.94). One-way ANOVA test showed the statistically significant differences between the groups (*p* = 0.000*). Tukey post-hoc test showed that there was a significant decrease in S1 and S2 as compared with C and S3. On the other hand, there was a significant decrease in all G groups as compared with C, S1 and S3.

#### Surface roughness (Ra)

Surface roughness (µm) of all tested groups means ± standard deviations values were presented in Table 5. The highest Ra mean value was recorded for S3 (0.522 ± 0.059). The lowest value was recorded for C (0.239 ± 0.019). One-way ANOVA test showed the statistically significant differences between the groups (*p* = 0.000*). Tukey post-hoc test showed that there was a significant increase in S groups as compared with C Group. Also, there was a significant increase in S2 and S3 as compared with S1. On the other hand, there was no significant increase in G groups as compared with C Group. But there was a significant decrease in G groups as compared with S Groups.

**Fig. 7:**
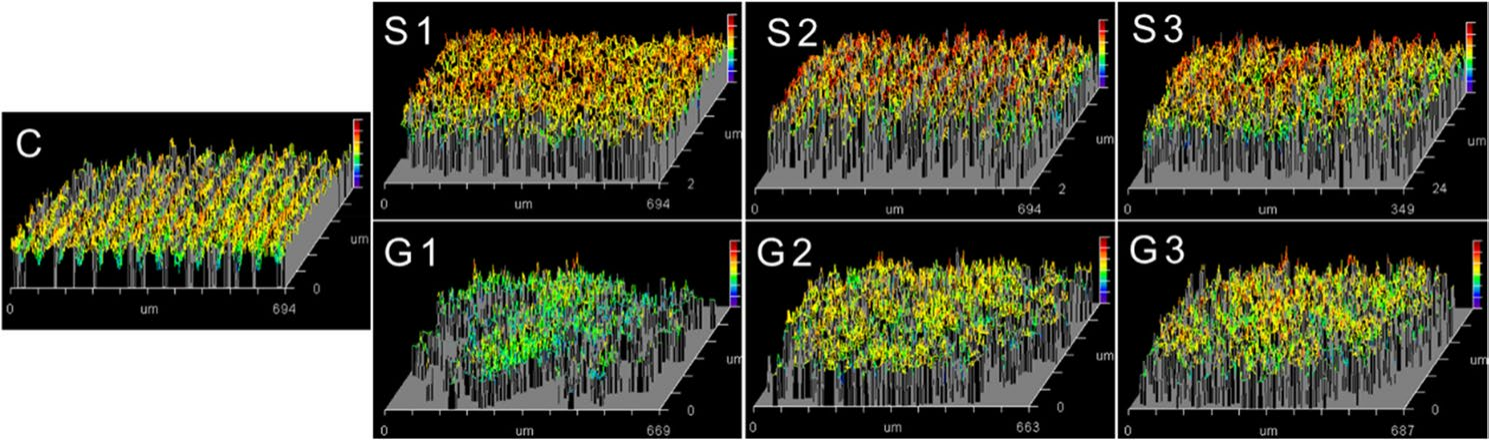
Optical profilometer micrographs showing surface roughness of the surfaces of all groups

#### Surface Vickers microhardness (HVN)

Vickers microhardness values (HVN) of all tested groups means ± standard deviations values were presented in Table 5. The highest HVN mean value was recorded for S1 (18.55 ± 2.1). The lowest value was recorded for G1 (11.8 ± 2). One-way ANOVA test showed the statistically significant differences between the groups (*p* = 0.000*). Tukey post-hoc test showed that there was a slight non-significant increase in S1 as compared with C but there was a significant decrease in S2 and S3 as compared with S1 and C. On the other hand, there was a significant decrease in G1 and G2 as compared with C. There was a gradual significant increase (*p* < 0.05) in G groups by increasing filler wt% (G1: 11.8 ± 2.0; G2: 14.7 ± 1.9; G3: 17.5 ± 1.6).

#### Flexural strength (σ)

Flexural strength (MPa) of all tested groups means ± standard deviations values were presented in Table 5. The highest σ mean value was recorded for S1 (73 ± 8.2). The lowest value was recorded for G1 (63.8 ± 6.5). One-way ANOVA test showed the statistically significant differences between the groups (*p* = 0.020*). Tukey post-hoc test showed that there was a slight non-significant increase in S1 and S2 as compared with C. But there was a gradual non-significant decrease (*p* > 0.05) in S groups by increasing filler wt% (S1: 73.0 ± 8.2; S2: 71.3 ± 4.2; S3: 65.6 ± 5.4). On the other hand, there was a slight non-significant decrease in G groups as compared with C group. But there was a significant decrease in G1 as compared with S1. There was a gradual non-significant increase (*p* > 0.05) in G groups by increasing filler wt% (G1: 63.8 ± 6.5; G2: 65.5 ± 5.5; G3: 66.1 ± 7.7).

#### Flexural modulus (E)

Flexural modulus (MPa) of all tested groups means ± standard deviations values were presented in Table 5. The highest E mean value was recorded for S1 (2427 ± 389). The lowest value was recorded for G1 (1740 ± 207). One-way ANOVA test showed the statistically significant differences between the groups (*p* = 0.000*). Tukey post-hoc test showed that there was a slight non-significant increase in S groups as compared with C group. But there was a gradual non-significant decrease (*p* > 0.05) in S groups by increasing filler wt% (S1: 2427 ± 389; S2: 2336 ± 199; S3: 2254 ± 372). On the other hand, there was a significant decrease in G1 and a non-significant decrease in G2 and G3 as compared with C. Also, there was a significant decrease in G1 and G2 as compared with corresponding S groups. There was a gradual non-significant increase (*p* > 0.05) for G groups by increasing filler wt% (G1: 1740 ± 207; G2: 1945 ± 160; G3: 2080 ± 214).

#### Fracture resistance (FR)

Fracture resistance (N) of all tested groups means ± standard deviations values were presented in Table 5. The highest fracture resistance mean value was recorded for S1 (988.1 ± 54). The lowest value was recorded for G1 (774.6 ± 38). One-way ANOVA test showed the statistically significant differences between the groups (*p* = 0.000*). Tukey post-hoc test showed that there was a significant increase in S1 as compared with C. For S groups, there was a non-significant decrease in S2 as compared with S1 and a significant decrease in S3 as compared with S1 and S2. On the other hand, there was a significant decrease in G groups as compared with C group. Also, there was a significant decrease in G1 and G2 as compared with corresponding S groups. There was a gradual non-significant increase (*p* > 0.05) in G groups by increasing filler wt% (G1: 774.6 ± 38; G2: 784.2 ± 89; G3: 852.6 ± 31).

### Spearman correlation

Spearman’s correlation analysis revealed relationships between filler fractions and the tested properties as presented in Table 6. For the inorganic filler fraction, a statistically significant negative correlation was observed with DC and WSL, while Ra showed a significant positive correlation. No significant correlations were detected with WSP, HVN, σ, E, or FR (*p* > 0.05). On the other hand, the organic filler fraction demonstrated significant correlations with several properties. DC exhibited a significant positive correlation, whereas WSP, Ra, E, and FR showed significant negative correlations *p* < 0.001). No statistically significant correlations were observed between with WSL, HVN, or σ (*p* > 0.05).

**Table 6:** Spearman correlation result for both filler fractions and tested properties

	MSN (Inorganic Filler Fraction)		MMA (Organic Filler Fraction)	
	*r*	Significance	*r*	Significance
DC%	-0.312	0.009*	0.374	0.001*
WSL (ug/mm3)	-0.379	0.001*	0.039	0.748
WSP (ug/mm3)	0.077	0.529	-0.639	0.000*
Ra (um)	0.577	0.000*	-0.494	0.000*
HVN	-0.215	0.073	0.036	0.770
σ (MPa)	-0.008	0.948	-0.215	0.073
E (MPa)	0.231	0.055	-0.437	0.000*
FR (N)	-0.020	0.867	-0.636	0.000*

An asterisk (*) indicates a statistically significant Spearman correlation (*p*< 0.05)

## Discussion

This study has evaluated the effect of simultaneous silanization and grafting reaction on the surface of MSN on their agglomeration. Also, the effect of modification of 3D-PPR with surface treated MSN, at different percentage, on its physicomechanical properties.

Incorporation of MSN into 3D printable resins was recommended at low concentrations (≤ 1% by weight) due to the low density of MSN compared to other metal oxides in addition to the very high surface area and surface charge of the inorganic nanoparticles [39, 40]. These particles tend to agglomerate at high concentrations with negative effects on physicomechanical properties of modified resin. Accordingly, low concentrations (0.25 wt % and 1 wt %) of MSN were selected. A higher concentration 3 wt % was added to evaluate the effect of surface modification of MSN on their agglomeration and subsequently on the physicomechanical properties of modified resin. The 3 wt% concentration was intentionally selected as a stress-test level. This concentration was considered sufficiently high to challenge filler dispersion stability, yet not excessively high to compromise resin processability, printability, or curing behavior. This balance enables a more rigorous evaluation of the effectiveness of the surface modification strategies.

According to previous studies [13, 41, 42], silane coupling agent treatment and surface grafting modification were the two most applied strategies to overcome the agglomeration of silica nanoparticles within the organic matrix. MSN was silanized by APTES because it is one of the most applied organosilanes due to its amino group termination which can be used as an intermediate layer for further functionalization. Silanization represents an adaptive mechanism that diminishes the surface energy of fillers, which protects nanoparticles from dissolving and enhances the interphases with resin, facilitating uniform dispersion as phases evolve. Typically, silane coupling agents adsorb on nanoparticle surfaces, which creates a chemisorbed silane layer and covalently binds to particle surfaces and then copolymerize with the methacrylic polymer matrix [43, 44].

The basic intermediate mechanism with MSN is the hydrolysis of APTES to form silanol (Si–O–H) and an amine functional group (NH_2_–). This activation leads to conjugation with the hydroxyl group of silica (Si–OH) followed by a condensation reaction to produce such chemical bonds between MSN and APTES (Si–O–Si). Further, the free radical organofunctional groups bond acrylic ester-based monomers MMA. For grafting, MMA was selected as a grafting monomer to be compatible and homogenous with MMA monomers of 3D printed resin PMMA [29, 43]. BET/BJH, FTIR, TGA and TEM images indicated that MSN were successfully modified with APTES and MMA.

BET/BJH analysis (Table 3) demonstrated a notable reduction in both surface area and pore volume after MMA grafting, suggesting partial pore blocking and limited nitrogen accessibility [45]. The apparent increase in average pore diameter after grafting does not represent real pore enlargement but rather arises from the inherent limitations of BJH analysis when a large fraction of small mesopores becomes inaccessible; under these conditions, the calculated pore size distribution is dominated by larger residual pores [46]. Nevertheless, the persistence of a measurable BET surface area and pore volume suggests that a fraction of the pore network remains partially accessible, despite extensive pore blocking induced by grafting [47, 48]. Overall, the combined changes in surface area, pore volume, and apparent pore diameter are fully consistent with successful polymer grafting within the mesoporous silica structure.

FTIR spectrum (Fig. 3A) and TGA thermogram (Fig. 4A) of S-MSN suggested successful silanization of MSN by APTES. The wide absorption peak at 1082 cm^− 1^ reflects siloxane (Si–O–Si) stretching mode. The characteristic peaks around 1638 cm^− 1^ belongs to N-H bending vibration and 1679 cm^− 1^ belongs to C–H stretching. Several minor bands around 2800 to 3000 cm^− 1^ are attributed to the N-H stretching vibration [49, 50]. In the TGA thermogram, weight loss below 200 °C is related to the presence of water or physically adsorbed APTES molecules on the surface of MSN. The second stage of thermal decomposition initiated at 200 °C is due to the decomposition of the APTES molecules. There was no significant weight loss at 217 °C (corresponding to APTES boiling point).

FTIR spectrum (Fig. 3B) and TGA thermogram (Fig. 4B) of GS-MSN indicated successful grafting of MMA on the surface of S-MSN. The absorption bands at 3439 cm^–1^, 2929 cm^–1^, 1720 cm^–1^, 1390 cm^–1^, 1246 cm^–1^ and 1150 cm^–1^ reflect N-H stretching, C-H stretching, C-O double bond stretching, C-N stretching, C-O stretching and Si–O–Si stretching, respectively. The absorption bands around 1717 cm^–1^ are collapsed bands due to the ester and carboxylic group C = O stretching vibrations [29, 51]. The FTIR spectrum displayed a weak aliphatic C = C band at approximately 1638 cm⁻¹, reflecting the effective consumption of methacrylate double bonds during the curing process. In the TGA thermogram, a small weight loss before 200 °C is related to the removal of water and the decomposition of the unreacted MMA molecules. In the next step, a large weight loss is observed in the range of 280–400 °C, which is attributed to the thermal decomposition of the bonded MMA on the surface of S-MSN [39, 52].

According to the difference between the weight losses of S-MSN and GS-MSN at the temperature range of 200–800 °C, the percentage of the bonded MMA on the surface of S-MSN was obtained as 31%. The obtained high grafting percentage of MMA onto S-MSN in this study indicates the choice of a suitable coupling agent that has a high reactivity with MMA. So, there was another effect of grafting by changing inorganic filler fraction inside each filler wt% as shown in Table 4 [19].

TEM images (Fig. 2) indicated that MSN were successfully modified with an APTES and MMA without altering the structure or size of MSN, the nanoparticles had a spherical porous shape with average size 25 nm, similar to initial unmodified particles surrounded with thin film. In addition, TEM images of GS-MSN (Fig. 2C) showed a less agglomeration between nanoparticles in comparison with S-MSN (Fig. 2B), which was directly reflected on nanoparticles distribution as shown in SEM images (Fig. 5).

SEM images (Fig. 5) of experimental groups with S-MSN showed clear increase in nanoparticles agglomeration and decrease in their distribution by increasing the filler wt%. The same is noticed for experimental groups with GS-MSN but in less degree specially at G3 as compared with S3 and both with highest filler wt%, which could reasonably be due to MMA grafting which creates a cloud structure chemically bonded and surrounding S-MSN. This modification decreased agglomeration of nanoparticles which subsequently improved the dispersion of fillers within the resin. In addition, this improved the compatibility and adhesion between GS-MSN containing MMA and MMA of 3D-PPR [53].

So according to the previous findings, the first null hypothesis that the simultaneous silanization and grafting reaction on the surface of MSN would have no effect on their agglomeration was rejected.

Physicomechanical properties of dental resin are an important factor that directly influences longevity of provisional restorations. In this study, DC results as shown in (Fig. 6) suggested that there were no significant differences in DC between control group and all experimental groups. But there was a slight non-significant decrease in DC by increasing filler wt% for S groups and G groups. This can be explained by increasing filler wt% into 3D-PPR led to increased resin viscosity, which might limit mobility of unreacted monomer molecules [19, 43, 54]. On the other hand, there was a significant increase in DC for each G group when compared with the corresponding S group with the same filler wt%. This increase may be related to organic fraction (31 wt%) of filler in G groups which is composed of heat-polymerized PMMA [39]. Heat-polymerized PMMA exhibits a significantly higher degree of conversion than photo-polymerized PMMA, owing to enhanced molecular mobility and more complete monomer-to-polymer transformation under elevated thermal energy, whereas light-activated systems remain limited by light penetration, oxygen inhibition and the layer-by-layer curing mechanism [55, 56]. These findings were well aligned with the significant associations identified through Spearman’s rank correlation analysis as shown in Table 6.

In dental restorations, solubility and water sorption are essential parameters to assess the resistance to surrounding oral fluid, due to expansion and hydrolytic degradation, ultimately influencing its clinical longevity. Intrinsically, polarity is an inherent feature of polymers that allows water molecules to penetrate and eventually affect the mechanical properties [43]. Our investigation, as shown in Table 5, revealed that with addition of S-MSN in low concentrations (0.25-1%) in S1 and S2 there was significant decrease in water sorption of modified resin as compared with unmodified one. This decrease can be explained as a direct reflection of the replacement of organic fraction by inorganic one. In addition to the benefit of modifying MSN with APTES, which reasonably improves hydrophobicity and diminishes water uptake [21, 57]. However, the addition of S-MSN in higher concentrations (3%) in S3 showed significant increase in water sorption as compared with low concentration in S1 and S2 but with comparable value as to unmodified resin. This behavior may be due to the water sorption properties of MSN itself in addition to their agglomeration with higher concentration as water sorption of modified resin is affected by the material homogeneity [36].

On the other hand, there was a significant decrease in water sorption of all G groups as compared with C, S1 and S3. This decrease can be explained by homogenous distribution of GS-MSN in G groups. In addition to the organic filler fraction (31 wt%), represented by the MMA-grafted layer, which is hydrophobic in nature and composed of heat-polymerized PMMA with inherently lower water sorption than 3D-printed resins. This has been attributed to the higher polarity and monomer reactivity of 3D-printed resins which results in a reduced degree of conversion. In addition to the method of printed building, which might permit water to seep between the printed layers [58]. This observation corroborated the significant associations identified through Spearman’s correlation outcomes as shown in Table 6.

The relationship between degree of conversion, water sorption, and solubility with PMMA resin could be ascribed to several factors. As solubility depends mainly on the amount of converted monomer and reflects leaching of unreacted resin. This happens because of monomer entrapment during polymerization within polymer chains or micropores as a result of inadequate handling techniques or agglomeration of fillers [43, 59]. So, there was a significant decrease in solubility for all S and G groups as compared with the control group as listed in Table 5. This reduction of solubility can be hypothesized by the addition of S-MSN or GS-MSN which may have reacted with the residual monomers.

Surface roughness is an important characteristic that should be considered when fabricating dental restorations. Indeed, a very rough surface is significantly correlated with poor aesthetics, superficial staining, gingival inflammation, and bacterial plaque accumulation. Surface roughness measurements as shown in (Fig. 7) and Table 5 revealed that there was a significant increase in surface roughness in S groups as compared with C Group. This increase is a direct reflection to the agglomeration of nano fillers on the surface and concentration in confined areas rather than uniformity dispersion over the surface which is affected by increase in their percentage [18]. So, there was a significant increase in surface roughness in S2 and S3 with higher filler content (1–3 wt%) as compared with S1 with low filler content (0.25 wt%). As surface roughness has been closely associated with plaque accumulation, increased staining susceptibility, and unfavorable soft tissue responses, the concomitant increase in surface roughness may introduce potential clinical drawbacks if not properly controlled. Therefore, the present findings highlight the importance of achieving a balanced material design in which mechanical reinforcement and functional performance are optimized without compromising surface characteristics that are critical for long-term clinical acceptability. On the other hand, there was no significant increase in surface roughness in G groups as compared with C Group. This can be explained by the positive effect of grafting on homogeneous dispersion of fillers into resin. So, there was a significant decrease in surface roughness in G groups as compared with S Groups. These findings were in agreement with the observed Spearman’s rank correlation analysis as shown in Table 6.

Surface hardness is related to many factors such as depth of polymerization, organic / inorganic ratio and fillers type, size and dispersion. Nano-sized fillers have more contact area with resin than larger fillers; therefore, well dispersion of fillers helped significantly to increase material hardness. So, surface hardness increased proportionately not only to the inorganic filler content but also to well dispersion of fillers [60]. Surface hardness measurements as listed in Table 5 revealed that there was a slight non-significant increase in microhardness in S1 group as compared with C group but there was significant decrease in microhardness in S2 and S3 as compared with S1 and C groups. This reduction again is a direct reflection to agglomeration of nano fillers on the surface and concentration in confined areas rather than uniformity dispersion over the surface which was affected by increase in their percentage [18]. On the other hand, there was a significant decrease in microhardness in G1 and G2 groups as compared with C Group. This can be explained by the organic fraction (31wt%) of fillers in G groups which has lower effect on microhardness as compared with inorganic fraction [39]. So, there was a gradual significant increase in microhardness for G groups by increasing inorganic filler wt%. In contrast to S groups, G groups had a gradual significant increase in microhardness by increasing filler wt%. This can be explained by the positive effect of grafting on homogeneous dispersion of fillers inside the polymer network.

Clinically, internal microfractures, voids, porosity, and crack propagation are the major causes behind restoration failure. Flexural strength, flexural modulus and fracture resistance are indispensable properties that describe the capacity of dental restorations to absorb or withstand load or initial crack [43]. Flexural strength, flexural modulus and fracture resistance results as listed in Table 5 revealed that by comparing with C group, there was a slight non-significant increase in flexural strength in S1 and S2, a slight non-significant increase in flexural modulus in S groups and a significant increase in fracture resistance in S1. This increase can be explained as a direct reflection to the addition of inorganic filler. However, there was a gradual non-significant decrease in flexural strength and flexural modulus in S groups by increasing filler wt% and there was a non-significant decrease in fracture resistance in S2 as compared with S1 and significant decrease in S3 as compared with S1 and S2. This reduction can be explained by agglomeration of nano fillers and concentration in confined areas rather than uniformity dispersion inside the polymer network as fillers at a high loading tended to agglomerate and behaved as local stress concentration areas or crack-initiation sites that hindered polymer chain mobility and then jeopardized mechanical properties [61].

On the other hand, for flexural strength, there was a slight non-significant decrease in G groups as compared with C group and a significant decrease in G1 as compared with the corresponding S group. For flexural modulus, there was a significant decrease in G1 and a non-significant decrease in G2 and G3 as compared with C group and a significant decrease in G1 and G2 as compared with the corresponding S groups. For fracture resistance, there was a significant decrease in G groups as compared with C group and a significant decrease in G1 and G2 as compared with corresponding S groups. This reduction can be explained by the organic fraction (31%) of fillers, represented by the MMA-grafted layer, in G groups which has a lower effect on mechanical properties as compared with inorganic fraction as further demonstrated by Spearman’s rank correlation analysis in Table 6. So, it was noticeable that G1 had the lowest values as it had the lowest inorganic filler wt% within all experimental groups.

For flexural strength, flexural modulus and fracture resistance, there was a gradual non-significant increase in G groups by increasing inorganic filler wt% and in contrast to S groups, G groups had a gradual non-significant increase by increasing the filler wt%. This increase in G groups can be explained by the positive effect of grafting on homogeneous dispersion of fillers inside the polymer network.

So, according to the previous findings, the second null hypothesis that the modifying 3D-PPR with the simultaneous silanized and grafted MSN would have no effect on their physicomechanical properties was rejected.

Finally, it should be mentioned that this study was limited to just one brand of 3D-PPR, one printing technology and one nanoparticle in vitro. The lack of aging, thermocycling, fatigue testing and simulated oral environment, including factors like saliva, bacteria, and masticatory load could limit the interpretation of this study’s results and the durability-related outcomes. Also, we recommend retesting the specimens after some time-intervals to evaluate the effect of aging on phisycomechanical properties of modified resin by some executive limitations. Thus, longer follow-ups and aging processes such as thermal cycling and cyclic loading are suggested for further studies on similar issues. In addition, more characteristics such as translucency and ions rechargeability can be investigated in further studies. For future prospective, a comparative study with the current gold standard conventional heat cured materials should be considered to provide a full image about potential replacement, as this work showed promising results and high feasibility to be applied clinically. Another limitation of this study was the organic filler fraction inside each filler wt% after grafting which had a misleading result about filler wt%.

## Conclusion

Within the limitations of this study, it was concluded that:

1. The simultaneous surface silanization and grafting reaction on the surface of MSN decreased their agglomeration and enhanced their distribution within 3D-PPR.
2. The modified 3D-PPR with GS-MSN showed a gradual enhancement in physicomechanical properties by increasing filler wt%, in contrast to modification with S-MSN which showed a gradual decrease in physicomechanical properties.
3. The newly developed 3D-PPR may have the superior potential for increasing filler wt% with better distribution of fillers.

## Data Availability

On reasonable request, the datasets utilized and/or analyzed during the present study are accessible from the corresponding author.

## Abbreviations

3D: Three-dimensional
MSN: Mesoporous silica nanoparticles
3D-PPR: 3D-printable provisional resin
APTES: (3-Aminopropyl) triethoxysilane
MMA: Methyl methacrylate
S-MSN: Silanized mesoporous silica nanoparticles
GS-MSN: Grafted silanized mesoporous silica nanoparticles
PMMA: Polymethyl methacrylate
SN: Silica nanoparticles
BPO: Benzoyl peroxide
SEM: Scanning electron microscopy
TEM: Transmission Electron microscopy
FTIR: Fourier transformed infrared spectroscopy
XRD: X-Ray diffraction analysis
TGA: Thermogravimetric analysis
BET: Brunauer–Emmett–Teller theory
BJH: Barrett–Joyner–Halenda
DC: Degree of conversion
WSL: Solubility
WSP: Water sorption
Ra: Surface roughness average
HVN: Vickers’ hardness number
σ: Flexural strength
E: Flexural modulus
FR: Fracture resistance
DPL: Digital Light Processing
STL: Stereolithography
